# Peer Review of “Information Technology Ambidexterity, Digital Dynamic Capability, and Knowledge Processes as Enablers of Patient Agility: Empirical Study”

**DOI:** 10.2196/34113

**Published:** 2021-12-06

**Authors:** Laura Taraboanta

**Affiliations:** 1 Click Therapeutics New York, NY United States

**Keywords:** IT ambidexterity, dynamic capabilities, digital dynamic capability, knowledge processes, patient agility, hospitals, information sciences, information technology, digital health, health care, digital transformation, research models

*This is a peer review of “Information Technology Ambidexterity, Digital Dynamic Capability, and Knowledge Processes as Enablers of Patient Agility: Empirical Study”*

## Round 1 Review

### General Comments

Well thought out study design [1] with specific hypotheses and methods of analysis spelled out. Interesting conclusions drawn out that would be fruitful for further discussion and analysis to replicate on a broader sample of hospital systems outside of the current reviewed sites.
